# Dysregulation of Immune Response Mediators and Pain-Related Ion Channels Is Associated with Pain-like Behavior in the GLA KO Mouse Model of Fabry Disease

**DOI:** 10.3390/cells11111730

**Published:** 2022-05-24

**Authors:** Marlene Spitzel, Elise Wagner, Maximilian Breyer, Dorothea Henniger, Mehtap Bayin, Lukas Hofmann, Daniela Mauceri, Claudia Sommer, Nurcan Üçeyler

**Affiliations:** 1 Department of Neurology, University of Würzburg, 97080 Würzburg, Germany; spitzel_m@ukw.de (M.S.); wagner_e4@ukw.de (E.W.); breyer_m@ukw.de (M.B.); hennigerdorothea@gmail.com (D.H.); mehtap.bayin@gmail.com (M.B.); lukas.hofmann1@gmx.de (L.H.); sommer@uni-wuerzburg.de (C.S.); 2 Department of Neurobiology, Heidelberg University, 69120 Heidelberg, Germany; mauceri@nbio.uni-heidelberg.de

**Keywords:** Fabry disease, globotriaosylceramide, inflammation, macrophages, cytokines, ion channels, flotillin-1 lipid rafts, pain-associated behavior, mouse model

## Abstract

Fabry disease (FD) is a rare life-threatening disorder caused by deficiency of the alpha-galactosidase A (GLA) enzyme with a characteristic pain phenotype. Impaired GLA production or function leads to the accumulation of the cell membrane compound globotriaosylceramide (Gb3) in the neurons of the dorsal root ganglia (DRG) of FD patients. Applying immunohistochemistry (IHC) and quantitative real-time polymerase chain reaction (qRT PCR) analysis on DRG tissue of the GLA knockout (KO) mouse model of FD, we address the question of how Gb3 accumulation may contribute to FD pain and focus on the immune system and pain-associated ion channel gene expression. We show a higher Gb3 load in the DRG of young (<6 months) (*p* < 0.01) and old (≥12 months) (*p* < 0.001) GLA KO mice compared to old wildtype (WT) littermates, and an overall suppressed immune response in the DRG of old GLA KO mice, represented by a reduced number of CD206^+^ macrophages (*p* < 0.01) and lower gene expression levels of the inflammation-associated targets interleukin(IL)1b (*p* < 0.05), IL10 (*p* < 0.001), glial fibrillary acidic protein (GFAP) (*p* < 0.05), and leucine rich alpha-2-glycoprotein 1 (LRG1) (*p* < 0.01) in the DRG of old GLA KO mice compared to old WT. Dysregulation of immune-related genes may be linked to lower gene expression levels of the pain-associated ion channels calcium-activated potassium channel 3.1 (KCa3.1) and transient receptor potential ankyrin 1 channel (TRPA1). Ion channel expression might further be disturbed by impaired sphingolipid recruitment mediated via the lipid raft marker flotillin-1 (FLOT1). This impairment is represented by an increased number of FLOT1^+^ DRG neurons with a membranous expression pattern in old GLA KO mice compared to young GLA KO, young WT, and old WT mice (*p* < 0.001 each). Further, we provide evidence for aberrant behavior of GLA KO mice, which might be linked to dysregulated ion channel gene expression levels and disturbed FLOT1 distribution patterns. Behavioral testing revealed mechanical hypersensitivity in young (*p* < 0.01) and old (*p* < 0.001) GLA KO mice compared to WT, heat hypersensitivity in young GLA KO mice (*p* < 0.001) compared to WT, age-dependent heat hyposensitivity in old GLA KO mice (*p* < 0.001) compared to young GLA KO mice, and cold hyposensitivity in young (*p* < 0.001) and old (*p* < 0.001) GLA KO mice compared to WT, which well reflects the clinical phenotype observed in FD patients.

## 1. Introduction

Fabry disease (FD), a rare X-linked multiorgan disorder, develops based on >900 known mutations in the alpha-galactosidase A (*GLA*) gene coding for the homonymous GLA enzyme [1], which is part of the cellular lipid metabolism. GLA is located within lysosomes and degrades the glycosphingolipid globotriaosylceramide (Gb3) [2]. In FD, GLA production or function is impaired by mutations within the coding gene sequence, leading to Gb3 accumulation in several cell types, including kidney cells, endothelial cells, and neurons [3,4]. FD patients suffer from nephropathy, cardiomyopathy, and cerebral stroke, contributing to an overall reduced life expectancy [5]. The peripheral nervous system is involved in the form of acral burning pain that may be triggered by heat, fever, and physical activity [4].

The pathophysiology linking Gb3 accumulation and FD pain development is still not fully understood. In animal studies, a potential direct impact of Gb3 on ion channel expression and function in murine dorsal root ganglion (DRG) neurons was shown, providing evidence for a potential contribution to FD pain [6,7]. Further, the involvement of altered immune response mechanisms in FD pathophysiology was suggested based on animal [8,9] and human studies [10,11]. In particular, clinical studies provide evidence for the swelling and consequent malperfusion of patients’ DRG when assessed using magnetic resonance imaging (MRI) [10]. This may cause local inflammation [12,13], which further may trigger FD pain.

In FD research, the GLA knockout (KO) mouse model is widely used for animal studies. This mouse model is based on a constitutive ablation of the *GLA* gene [14], which mimics closely the clinical and cellular phenotype seen in FD patients [7,15,16,17]. 

We hypothesize that Gb3 accumulations in murine DRG neurons lead to inflammatory responses causing the immune cell infiltration of T-cells and macrophages and inflammation-associated gene expression alterations. Further, dysregulated immune responses may lead to altered gene expression levels of selected pain-associated ion channels, such as the calcium-activated potassium channel 3.1 (KCa3.1) [18,19,20], the transient receptor potential ankyrin 1 channel (TRPA1) [21], the transient receptor potential melastatin 8 channel (TRPM8) [22], the voltage-gated calcium channel 2.2 (CaV2.2) [23], and the voltage-gated sodium channel 1.8 (NaV1.8) [24], further inducing nocifensive behavior in the GLA KO mouse model. Ion channel expression might also be influenced by rearrangement processes within the cellular membrane and recruitment processes of several receptors and channels from the nucleus into the cellular membrane mediated via lipid raft components including flotillin-1 (FLOT1) [25,26].

To test this hypothesis, we have investigated the potential interplay of neuronal Gb3, local immune response, ion channel gene expression, and pain-like behavior in the GLA KO mouse model of FD.

## 2. Materials and Methods

### 2.1. Animal Groups

Our experiments on mice used for behavioral and molecular analyses were approved by the Bavarian State authorities (Regierung von Unterfranken #54/12; #1052-22). Mice were kept in the animal facilities of the Department of Neurology and of the Centre for Experimental Molecular Medicine (Zentrum für Experimentelle Molekulare Medizin, ZEMM), University of Würzburg, Germany. Commercially available standard chow and water were offered to the animals *ad libitum*. We investigated homozygous GLA KO and WT littermate mice bred with an identical genetic background. We performed genotype analysis on every newborn mouse included in our studies using the Taq PCR Master Mix Kit (Qiagen, Hilden, Germany) and the following primers: oIMR5947, AGGTCCACAGCAAAGGATTG; oIMR5948, GCAAGTTGCCCTCTGACTTC; oIMR7415, GCCAGAGGCCACTTGTGTAG (Invitrogen, Carlsbad, CA, USA). To properly reflect the age-dependent progression of FD, we stratified our mice for age (young mice < 6 months, old mice ≥ 12 months).

### 2.2. Tissue Collection

Mice were euthanized in deep isoflurane anesthesia (CP-Pharma, Burgdorf, Germany) and exsanguinated. We dissected lumbar (L)3-5 DRG, while L3 and L5 were used for quantitative real-time polymerase chain reaction (qRT PCR) analysis and L4 was used for immunohistochemistry (IHC). L3 and L5 DRG were collected in tubes and flash-frozen in liquid nitrogen-cooled 2-methylbutane (Carl Roth, Karlsruhe, Germany). L4 DRG were embedded in optimal cutting temperature medium (TissueTek^®^, Sakura Finetek, Staufen, Germany) and flash-frozen in liquid nitrogen-cooled 2-methylbutane. Tissue was stored at −80 °C until further processing.

### 2.3. Immunohistochemistry

L4 DRG were used for preparing 10-µm-thin cryosections cut with a cryostat (Leica Microsystems, Wetzlar, Germany). Three sections per animal were collected on each slide and stored at −20 °C until further processing. For 3,3′-diaminobenzidine (DAB) staining, cryosections were fixed in acetone (Sigma-Aldrich, St. Louis, MO, USA) for 10 min at −20 °C and blocked with 10% Bovine Serum Albumin/Tris-hydroxymethyl-aminomethane (BSA/Tris, Sigma-Aldrich, St. Louis, MO, USA) for 30 min at room temperature (RT). Primary antibodies included anti-CD11b (rat anti-mouse, 1:250, MCA711, Bio-Rad (formerly AbD Serotec), Hercules, CA, USA) as a pan-macrophage marker and anti-CD3 (rat anti-mouse, 1:100, MCA1477, Bio-Rad, Hercules, CA, USA) as a T-cell-specific marker. Primary antibodies were diluted in 1% BSA/Tris and 2% milk powder (Sigma-Aldrich, St. Louis, MO, USA) at the respective concentration and incubated overnight at 4 °C. After three washing steps with Tris for 5 min each, cryosections were incubated with methanol and 30% hydrogen peroxide (H_2_O_2_) (both Sigma-Aldrich, St. Louis, MO, USA) for 20 min at RT following incubation with the secondary antibody diluted in 1% BSA/Tris and 2% milk powder for 30 min at RT. The secondary antibody contained a mouse absorbed immunoglobulin G (IgG) (rabbit anti-rat IgG (H + L), 1:50; BA-4001, Vector Laboratories, Inc., Burlingame, CA, USA). Sections were washed three times for 5 min each and treated with the Avidin/Biotin Blocking Kit and DAB Substrate Kit, Peroxidase (with Nickel) (both Vector Laboratories, Inc., Burlingame, CA, USA) according to the manufacturer’s instructions. Thereafter, sections were incubated with hematoxylin–eosin (Sigma-Aldrich, St. Louis, MO, USA) for 30 s at RT to visualize nuclei and were dehydrated with an ascending alcohol row and two washing steps with xylole (Carl Roth, Karlsruhe, Germany) for 10 min at RT. Sections were mounted with Vitro-Clud^®^ (R. Langenbrick GmbH, Emmendingen, Germany) and stored at RT until image acquisition.

For immunofluorescent staining, cryosections were fixed in acetone for 10 min at −20 °C and blocked with 10% Bovine Serum Albumin/Phosphate-Buffered Saline (BSA/PBS, Sigma-Aldrich, St. Louis, MO, USA) for 30 min at RT, followed by incubation with the respective primary antibodies diluted in 1% BSA/PBS and 0.1% saponin (Sigma-Aldrich, St. Louis, MS, USA) overnight at 4 °C. Primary antibodies included anti-CD80 (anti-mB7-1 goat anti-mouse, 1:50, AF740, R&D Systems, Minneapolis, MN, USA) as an M1 macrophage marker, anti-CD206 (rat anti-mouse, 1:100, MCA2235GA, Bio-Rad, Hercules, CA, USA) as an M2 macrophage marker, and anti-F4/80 (rat anti-mouse, 1:300, MCA497R, Bio-Rad, Hercules, CA, USA) as a pan-macrophage marker. For FLOT1 distribution analysis, we used an anti-FLOT1 antibody (rabbit anti-mouse, 1:150, F1180, Sigma-Aldrich, St. Louis, MO, USA). Additionally, we used Shiga toxin 1, subunit B (STxB, Sigma Aldrich, St. Louis, MO, USA) coupled with Alexa Fluor 555 (Thermo Fisher Scientific, Waltham, MA, USA) (STxB::555, 1:5000, five µg/µL STxB was conjugated with Alexa Fluor 555 using Zebra spin desalting columns (Thermo Fisher Scientific, Waltham, MA, USA)) to visualize Gb3 deposits in DRG cryosections and anti-PGP9.5 (rabbit anti-mouse, 1:100, 516-3344, Zytomed Systems GmbH, Berlin, Germany) as a neuronal marker. Next, cryosections were washed three times for 5 min each with PBS and incubated with the respective secondary antibodies diluted in 1% BSA/PBS for 2 h at RT. Secondary antibodies included Cy3-labeled donkey anti-goat IgG for CD80 staining, donkey anti-rat IgG for CD206 staining, goat anti-rat IgG for F4/80 staining, Cy3- and 488-labeled donkey anti-rabbit IgG for FLOT1 staining, and Alexa Fluor 647-conjugated donkey anti-rabbit IgG for PGP9.5 staining (all 1:400, Jackson Immuno Research Laboratories Inc., Bar Harbor, ME, USA). The incubation was followed by three washing steps with PBS for 5 min each. Cryosections were mounted with VECTASHIELD^®^ containing 4′,6-diamidino-2-phenylindole (DAPI, Vector Labs, Burlingame, CA, USA) for nuclear staining and the coverslips were sealed with CoverGrip™ (Biotium Inc., Fremont, CA, USA). Fluorescence preparations were stored at 4 °C in a dark place until image acquisition to avoid bleaching.

### 2.4. Bright Field and Fluorescence Microscopy

For bright field image acquisition of DAB staining on DRG cryosections, we used a Leica DMI IL LED inverted microscope with a DMC2900 CCD camera (Leica Microsystems, Wetzlar, Germany). Images were acquired with the Leica Application Suite X software (Leica Microsystems, Wetzlar, Germany) at 20× magnification and were used for cell counting of CD11b^+^ and CD3^+^ cells in DRG sections. For fluorescence image acquisition, we used the fluorescence microscope Zeiss Imager M.2 with an Apotome 2 device for structured illumination, Colibri 7 LED as a light source, and an Axiocam 506 mono CCD camera (all Zeiss, Oberkochen, Germany), and the fluorescence microscope Zeiss Axiophot 2 (Zeiss, Oberkochen, Germany) equipped with a SPOT INSIGHT^TM^ 4.0 Mp Color Camera and the SPOT Advanced Software (both SPOT imaging, Sterling Heights, MI, USA). Images were acquired with the Zeiss ZEN Blue edition software at 20× magnification. 

### 2.5. Image Analysis 

For DAB staining, CD11b^+^ macrophages and CD3^+^ T cells were counted live on the Leica bright field microscope at 20× magnification. For fluorescent staining, CD80^+^ M1 macrophages, CD206^+^ M2 macrophages, and F4/80^+^ macrophages were counted on previously acquired images at 20× magnification with the Zeiss fluorescence microscope. The investigator was blinded to the genotype and age of used animals. Three cryosections per animal were evaluated. Counted cells were marked and the cell body rich area (CBRA, according to [10]) of DRG cryosections was measured using ImageJ Fiji software [27]. Counted cells were normalized to the CBRA. 

For fluorescent Gb3 staining, images with STxB::555 and PGP9.5 double staining of murine DRG were acquired at 20× magnification with the Zeiss fluorescence microscope. The investigator was blinded to the genotype and age of the investigated animals. Three cryosections per animal were used for the analysis. The pan-neuronal marker PGP9.5 was applied to determine the CBRA with the Fiji software. The amount of Gb3 accumulation within DRG cryosections was determined indirectly within the CBRA by measuring the STxB::555 mean signal intensity using Fiji software. Mean intensity measurements were normalized to the investigated WT groups. Ratios of mean intensity values were calculated as GLA KO/WT.

For fluorescent FLOT1 distribution analysis, images with FLOT1 staining of murine DRG were acquired with the Zeiss fluorescence microscope. The total number of neurons per DRG cryosection and DRG neurons with a positive membranous FLOT1 signal were quantified by an investigator blinded to the genotype and age of used mouse groups. FLOT1^+^ DRG neurons were normalized to the total number of DRG neurons per section.

### 2.6. Gene Expression Analysis

Frozen L3 and L5 DRG samples were transferred into 700 µL of QIAzol Lysis Reagent (miRNeasy mini Kit, Qiagen, Hilden, Germany) and processed with the Polytron PT 3100 homogenizer (Kinematica, Luzern, Switzerland). Total RNA isolation was performed using the miRNeasy mini Kit (Qiagen, Hilden, Germany) following the manufacturer’s instructions. Then, 250 ng total RNA was reverse-transcribed using TaqMan^®^ Reverse Transcription Reagents (Applied Biosystems, Darmstadt, Germany).

To screen for inflammation-associated targets, we used standard (0.2-mL) TaqMan^®^ Array Plates (TaqMan^®^ Array Mouse Immune Response, Applied Biosystems, Darmstadt, Germany) preloaded with selected inflammation-associated gene expression assays (Table S1) and loaded every well with 10 µL cDNA pooled out of 6 animals per genotype and 10 µL TaqMan^®^ Fast Advanced Mastermix (Applied Biosystems, Darmstadt, Germany).

For single target validation analysis, we loaded 3.5 µL of cDNA per sample and 6.5 µL TaqMan^®^ Fast Advanced Mastermix (Applied Biosystems, Darmstadt, Germany) for duplex qRT PCR in triplets into standard (0.1-mL) TaqMan^®^ Array Plates (Applied Biosystems, Darmstadt, Germany) using specific mouse TaqMan^®^ Gene Expression Assays tagged with FAM-MGB reporter dye (all Gene Expression Assays purchased from Applied Biosystems, Darmstadt, Germany; respective Assay-IDs listed in Table 1). 

As an endogenous control, we used a TaqMan^®^ Gene Expression Assay for Ribosomal Protein L13a (RPL13A) (Mm01612986_gH) tagged with VIC-MGB reporter dye for the inflammation-associated targets and Glyceraldehyde-3-phosphate dehydrogenase (GAPDH) (Mm99999915_g1) tagged with VIC_MGB reporter dye for ion channel gene expression analysis. qRT PCR were run with a 96-well StepOnePlus™ Real-Time PCR System (Applied Biosciences, Darmstadt, Germany) at the following conditions: 2 min, 50 °C; 2 min, 95 °C; (3 s, 95 °C; 30 s, 60 °C) 40×. qRT PCR runs were acquired with the StepOne software v2.3 and relative gene expression analysis was calculated according to the ΔΔCt method.

### 2.7. Behavioral Testing

Behavioral testing was performed always at the same time of day by an experienced investigator blinded to the genotype. Tests included the von Frey test [28], investigating paw withdrawal thresholds by mechanical stimulation via calibrated von Frey filaments (Touch Test^TM^ Monofilaments, FMI GmbH, Seeheim-Ober Beerbach, Germany) according to the up-and-down-method; the Hargreaves test according to [29], investigating paw withdrawal latencies by heat stimulation via an infrared (IR) emitter (Plantar Test 7372, Ugo Basile S.R.I., Comerio, Italy), and cold sensitivity testing according to [30], investigating paw withdrawal latencies via cold stimulation with dry ice.

For mechanical sensitivity experiments, animals were placed within acryl glass boxes on a wire mesh. Hind paws were stimulated for up to 3 s with standardized von Frey filaments starting with a force of 0.6 g. Upon paw withdrawal, the next thinner filament was applied. If no paw withdrawal occurred, the next thicker von Frey filament was used. Six measurements per hind paw were performed and evaluated according to [28].

For heat sensitivity experiments, mice were placed within acrylic glass boxes on a glass plate. Hind paws were stimulated with a heat stimulus (25 IR) for up to 16 s to avoid tissue damage. Paw withdrawal latencies were measured 3 times per hind paw and the mean was calculated to determine the average paw withdrawal latency per animal [29]. 

For cold sensitivity experiments, animals were placed within the acrylic glass box set up, as described previously for the Hargreaves test. Hind paws were stimulated with dry ice for up to 4 s to avoid tissue damage. Paw withdrawal latencies were measured 3 times per hind paw and the mean was calculated to determine the average paw withdrawal latency per animal [30].

### 2.8. Statistics

Statistical analysis and graph design was performed with SPSS software Version 28.0.1.0 (IBM, Ehningen, Germany) and GraphPad PRISM Version 9.3.1 (GraphPad Software, Inc., La Jolla, CA, USA), respectively. First, data distribution was tested with the Kolmogorov–Smirnov test and with the Shapiro–Wilk test. For non-normally distributed data, the Mann–Whitney-U test was used, while, for normally distributed data, the independent t-test was used. For correlation analysis of M1 and M2 macrophages, Pearson’s correlation test was used due to normal data distribution. These data are plotted as a correlation graph with listed Pearson’s correlation coefficient r in the figure description. StxB::555 measurements, cell counting, and qRT PCR data are visualized as boxplots containing the median value with the upper and lower 25% and 75% quartile. STxB::555 ratio analysis is plotted as a bar graph depicting the mean value with standard deviation. Behavioral data of young and old WT mouse groups were pooled due to no age differences, as previously shown [31] (referred to as “pooled WT”). Significance was considered at a *p* value of <0.05.

## 3. Results

### 3.1. Higher Gb3 Load in DRG of Young and Old GLA KO Compared to WT Mice

**Figure 1:**
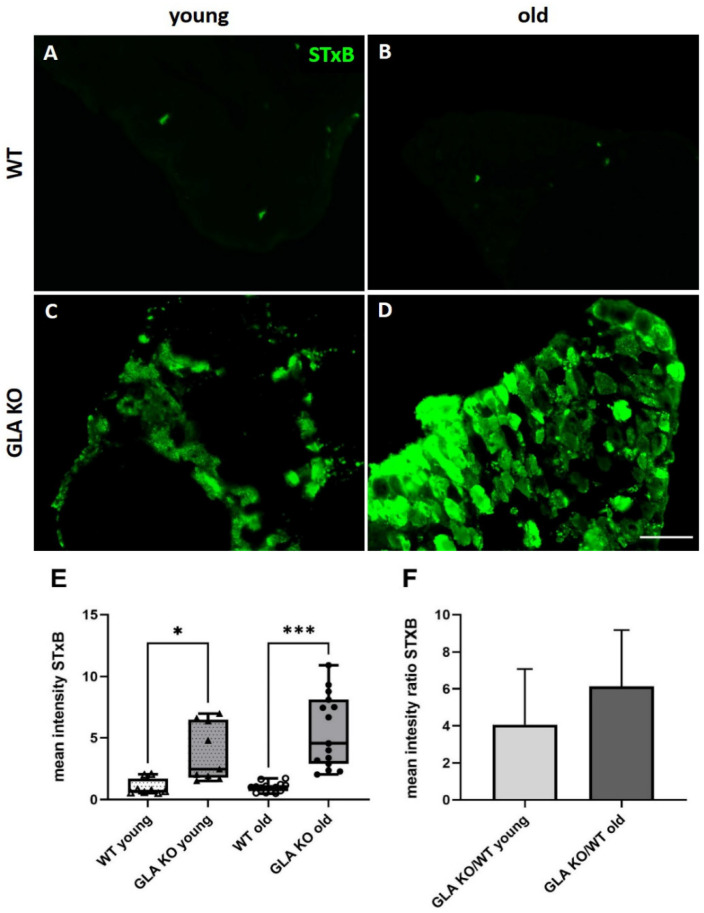
**Mean intensity of STxB::555 in murine DRG.** (**A**–**D**) Representative photomicrographs of Gb3 accumulations in DRG of young WT (**A**), old WT (**B**), young GLA KO (**C**), and old GLA KO mice (**D**) visualized with STxB::555 (green). (**E**) Intensity measurements of STxB::555 signal in murine DRG of young WT (△, n = 8), young GLA KO (▲, *n* = 9), old WT (◯, *n* = 14), and old GLA KO mice (●, *n* = 15). (**F**) Mean intensity GLA KO/WT ratio of STxB::555 signal for young and old murine DRG. Abbreviations: DRG = dorsal root ganglion; GLA KO = alpha-galactosidase A knockout; STxB = Shiga toxin 1, subunit B; WT = wildtype. Scale bar: 100 µm. * *p* < 0.05, *** *p* < 0.001.

First, we investigated the Gb3 load in young and old GLA KO and WT DRG using STxB::555 targeting Gb3 accumulations (Figure 1A–D). We found an average of 4-fold higher StxB::555 intensity in the DRG of young GLA KO compared to young WT (Figure 1E, *p* < 0.01). DRG of old GLA KO showed an average of 6-fold increased STxB::555 intensity compared to old WT (Figure 1E, *p* < 0.001). No inter-genotype differences were found for Gb3 load in young versus old WT mice (Figure 1E, *p* > 0.05). Indirect Gb3 load determination via STxB::555 intensity measurements revealed an average increase in the signal from 4-fold to 6-fold in young compared to old GLA KO DRG, respectively (Figure 1F, *p* > 0.05).

### 3.2. No Macrophage or T-Cell Infiltration in DRG of Old GLA KO Mice 

**Figure 2:**
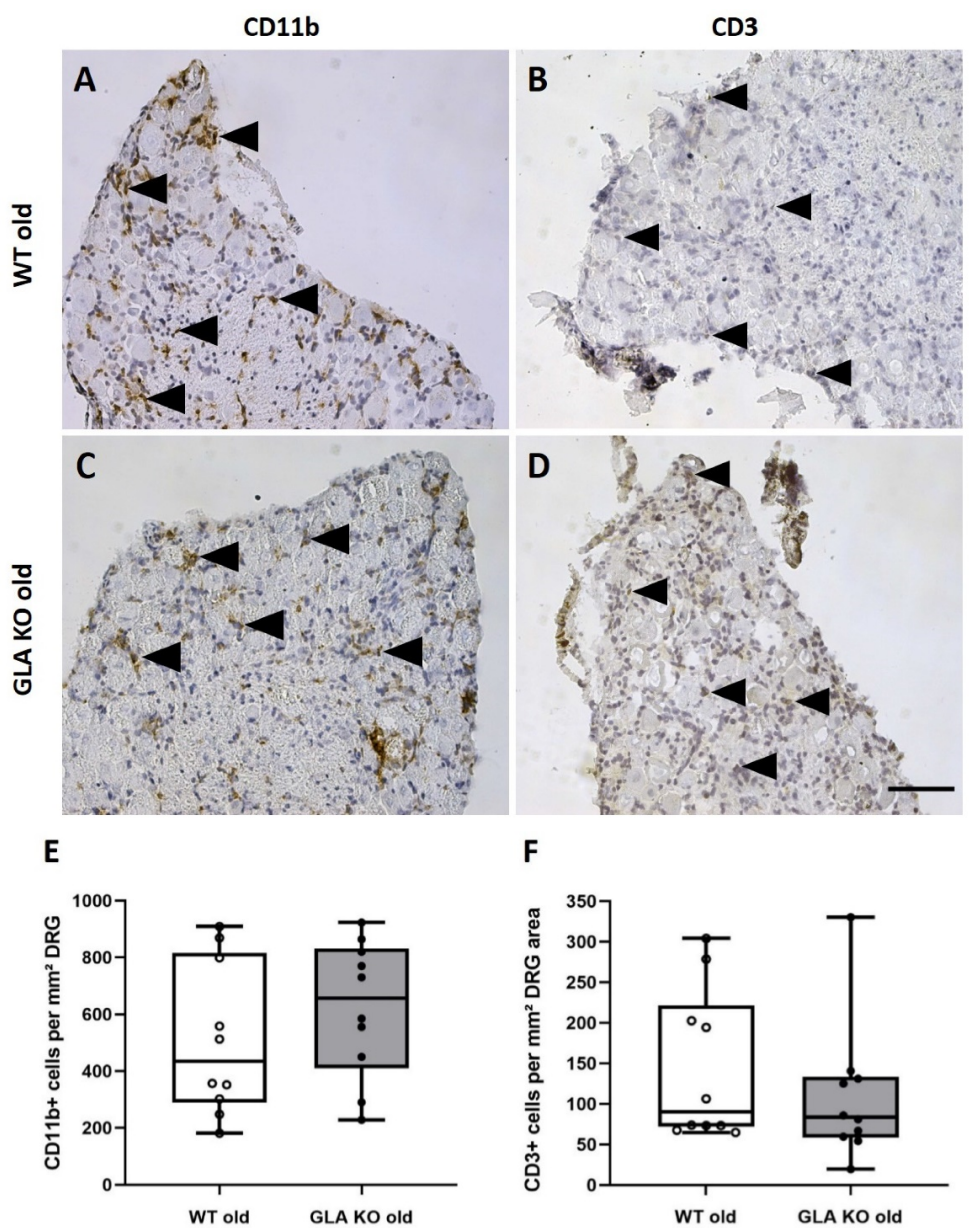
**Macrophage and T-cell infiltration in DRG of old WT and GLA KO mice.** (**A**–**D**) Representative photomicrographs of CD11b^+^ macrophages and CD3^+^ T-cells (arrowheads) in DRG of old WT ((**A**,**B**), respectively) and old GLA KO mice ((**C**,**D**), respectively). (**E**) Quantification of CD11b^+^ macrophages per mm² DRG area in old WT (◯, *n* = 10) and old GLA KO mice (●, *n* = 10). (**F**) Quantification of CD3^+^ T-cells per mm² DRG area in old WT (◯, *n* = 10) and old GLA KO mice (●, *n* = 10). Abbreviations: CD = Cluster of Differentiation; DRG = dorsal root ganglia; GLA KO = alpha-galactosidase A knockout; WT = wildtype. Scale bar: 100 µm.

Next, we asked whether macrophage and T-cell infiltrations occur as an immune reaction to increased Gb3 accumulation in mouse DRG. We analyzed the number of CD11b^+^ macrophages and CD3^+^ T-cells in DAB-stained DRG cryosections of old GLA KO and WT mice (Figure 2A–D). We found no difference in the number of CD11b^+^ (Figure 2E, *p* > 0.05) and CD3^+^ (Figure 2F, *p* > 0.05) cells per area DRG between old GLA KO and WT littermates.

### 3.3. Lower M2 Phenotype Macrophage Differentiation in DRG of Old GLA KO Compared to Old WT Mice

**Figure 3:**
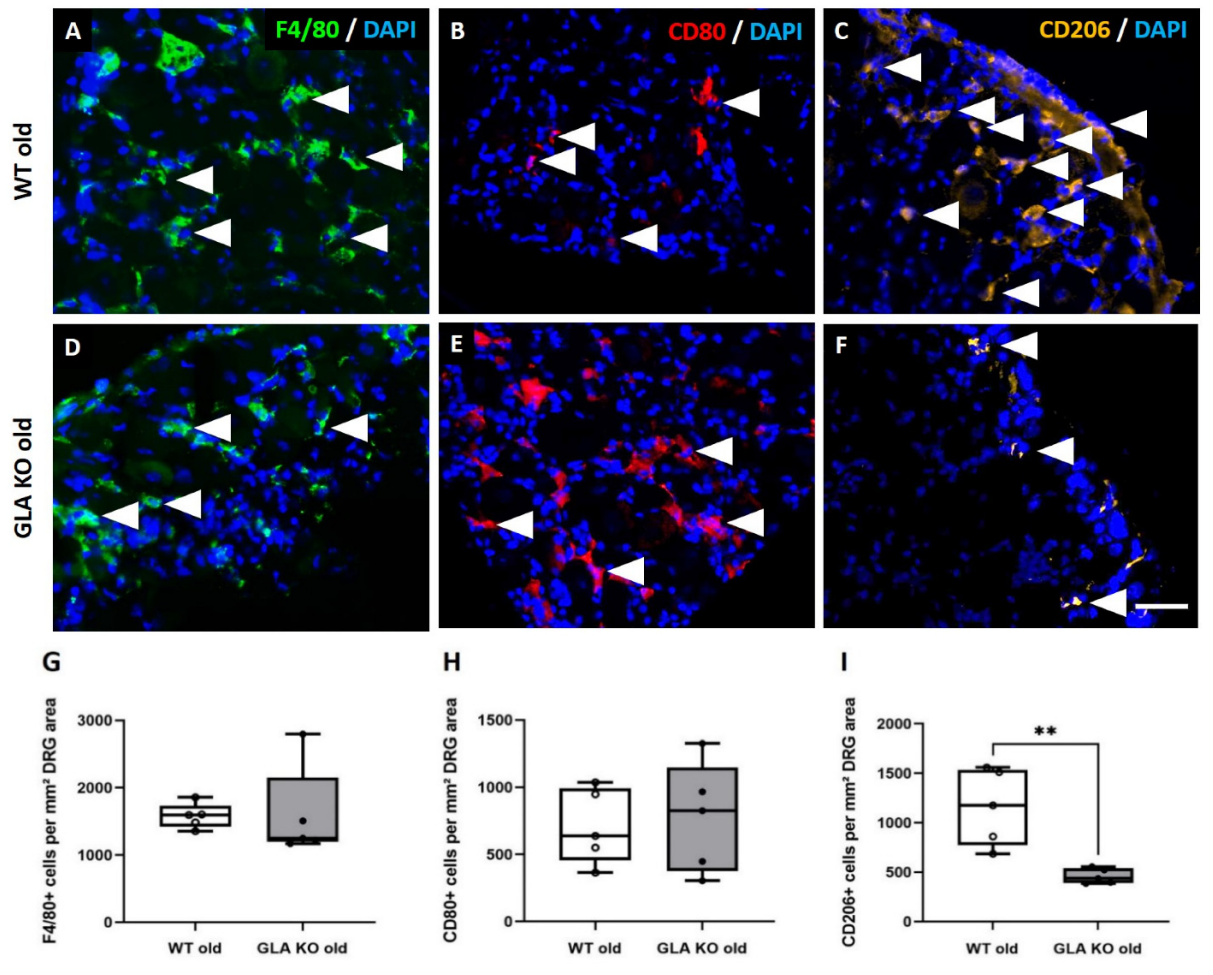
**Macrophage subtypes in DRG of old WT and GLA KO mice.** (**A**–**F**) Representative photomicrographs of F4/80^+^ (**A**,**D**), CD80^+^ (**B**,**E**), and CD206^+^ (**C**,**F**) macrophages (arrowheads) in DRG of old WT and old GLA KO mice. (**G**) Quantification of F4/80^+^ pan-macrophages per mm² DRG area in old WT (◯, *n* = 5) and old GLA KO mice (●, *n* = 5). (**H**) Quantification of CD80^+^ M1 macrophages per mm² DRG area in old WT (◯, *n* = 5) and old GLA KO mice (●, *n* = 5). (**I**) Quantification of CD206^+^ M2 macrophages per mm² DRG area in old WT (◯, *n* = 5) and old GLA KO mice (●, *n* = 5). Abbreviations: CD = Cluster of Differentiation; DAPI = 4′,6-diamidino-2-phenylindole; DRG = dorsal root ganglia; GLA KO = alpha-galactosidase A knockout; WT = wildtype. Scale bar: 25 µm. ** *p* < 0.01.

We further investigated the pro-inflammatory M1 and anti-inflammatory M2 macrophage phenotypes to assess whether the M1/M2 ratio differs between GLA KO and WT mice. We used F4/80 as a pan-macrophage (Figure 3A,D), CD80 as an M1-specific (Figure 3B,E), and CD206 as an M2-specific marker (Figure 3C,F) to determine the number of macrophage subtypes within the DRG of old GLA KO and WT mice. We found no differences for the numbers of F4/80^+^ (Figure 3G, *p* > 0.05) and CD80^+^ (Figure 3H, *p* > 0.05) macrophages between the DRG of old WT and GLA KO mice, while CD206^+^ immunoreactivity was markedly lower in the DRG of old GLA KO mice compared to old WT mice (Figure 3I, *p* < 0.01).

**Figure 4:**
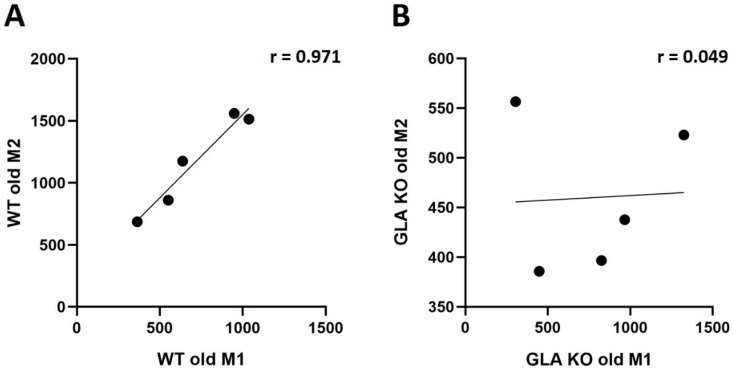
**M1/M2 ratio analysis in DRG of old WT and GLA KO mice.** (**A**) Pearson’s correlation analysis of M1 and M2 distribution in DRG of old WT mice (r = 0.971, *p* < 0.01). (**B**) Pearson’s correlation analysis of M1 and M2 distribution in DRG of old GLA KO mice (r = 0.049, *p* > 0.05). Abbreviations: DRG = dorsal root ganglia; GLA KO = alpha-galactosidase A knockout; r = Pearson’s correlation coefficient; WT = wildtype.

Correlation analysis (Figure 4) revealed no bidirectional polarization of M1/M2 macrophage subtypes in both genotypes. We found a positive correlation of M1 and M2 macrophages in DRG of old WT mice (Figure 4A, r = 0.971, *p* < 0.01), pointing towards a balanced ratio of M1 and M2 subtype distribution in the DRG of old WT mice, while, in the DRG of old GLA KO mice, we found no correlation between macrophage subtypes (Figure 4B, r = 0.049, *p* > 0.05).

### 3.4. Lower Expression of Inflammation-Associated Mediators IL1b, IL10, GFAP, and LRG1 in DRG of Old GLA KO Compared to Old WT Mice

**Figure 5:**
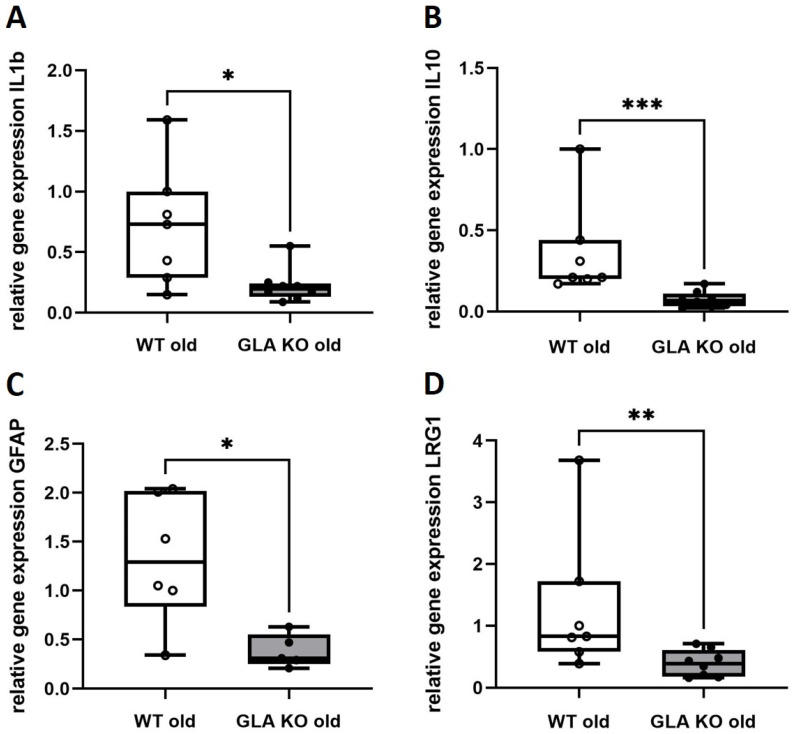
**qRT PCR analysis of inflammation-associated targets IL1b, IL10, GFAP, and LRG1.** (**A**–**D**) Relative gene expression of IL1b ((**A**), WT old n = 7, GLA KO old *n* = 8), IL10 ((**B**), WT old *n* = 7, GLA KO old *n* = 8), GFAP ((**C**), WT old *n* = 6, GLA KO old *n* = 5), and LRG1 ((**D**), WT old *n* = 7, GLA KO old *n* = 8) in DRG of old WT (◯) and old GLA KO mice (●). Abbreviations: DRG = dorsal root ganglia; GFAP = glial fibrillary acidic protein; GLA KO = alpha-galactosidase A knockout; IL = interleukin; LRG1 = leucine-rich alpha-2-glycoprotein 1; qRT PCR = quantitative real-time polymerase chain reaction; WT = wildtype. * *p* < 0.05, ** *p* < 0.01, *** *p* < 0.001.

Based on the lower M2 infiltration of anti-inflammatory macrophages, we asked if and which inflammation-associated gene targets were dysregulated in the DRG of GLA KO mice compared to WT. We performed a gene expression array analysis based on inflammation-associated targets to screen for potentially dysregulated markers (Table S1). Additionally, we included further gene targets based on literature research (Table S2). After array analysis and single qRT PCR validation, we found four downregulated target genes in the DRG of old GLA KO mice compared to old WT (Figure 5). These comprise the pro-inflammatory cytokine IL1b (Figure 5A, *p* < 0.05), the anti-inflammatory cytokine IL10 (Figure 5B, *p* < 0.001), the glial cell-specific marker GFAP (Figure 5C, *p* < 0.05), and the angiogenesis- and transforming growth factor beta (TGFb)-associated marker LRG1 (Figure 5D, *p* < 0.01).

### 3.5. Lower Ion Channel Gene Expression of KCa3.1 and TRPA1 in DRG of Old GLA KO Compared to Old WT Mice

**Figure 6:**
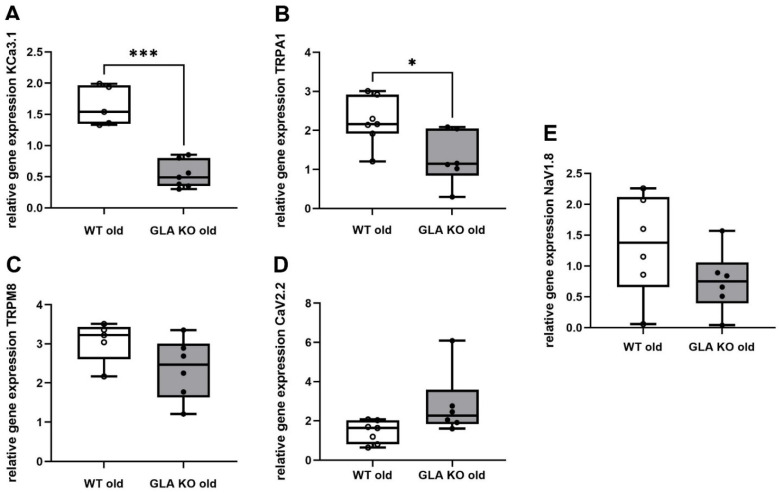
**qRT PCR analysis of pain-associated ion channels KCa3.1, TRPA1, TRPM8, CaV2.2, and NaV1.8.** (**A**–**E**) Relative gene expression of KCa3.1 ((**A**), WT old *n* = 5, GLA KO old *n* = 7), TRPA1 ((**B**), WT old *n* = 7, GLA KO old *n* = 6), TRPM8 ((**C**), WT old *n* = 5, GLA KO old *n* = 6), CaV2.2 ((**D**), WT old *n* = 7, GLA KO old *n* = 6), and NaV1.8 ((**E**), WT old *n* = 6, GLA KO old *n* = 6) in DRG of old WT (◯) and old GLA KO mice (●). Abbreviations: CaV2.2 = voltage-gated calcium channel 2.2; DRG = dorsal root ganglia; GLA KO = alpha-galactosidase A knockout; KCa3.1 = calcium-activated potassium channel 3.1; NaV1.8 = voltage-gated sodium channel 1.8; qRT PCR = quantitative real-time polymerase chain reaction; TRPA1 = transient receptor potential ankyrin 1; TRPM8 = transient receptor potential melastatin 8; WT = wildtype. * *p* < 0.05, *** *p* < 0.001.

To investigate whether the dysregulated inflammatory targets are associated with the gene expression of selected DRG neuronal pain-associated ion channels, we performed qRT PCR analysis for KCa3.1, TRPA1, TRPM8, CaV2.2, and NaV1.8 (Figure 6). We found lower expression of KCa3.1 (Figure 6A, *p* < 0.001) and TRPA1 (Figure 6B, *p* < 0.05) in DRG of old GLA KO mice compared to WT littermates, while TRPM8 (Figure 6C, *p* > 0.05), CaV2.2 (Figure 6D, *p* > 0.05), and NaV1.8 (Figure 6E, *p* > 0.05) did not show intergroup differences.

### 3.6. Old GLA KO Mice Show a Higher Number of FLOT1^+^ DRG Neurons Displaying a Membranous Distribution Pattern Compared to Young WT and GLA KO and Old WT Mice

**Figure 7:**
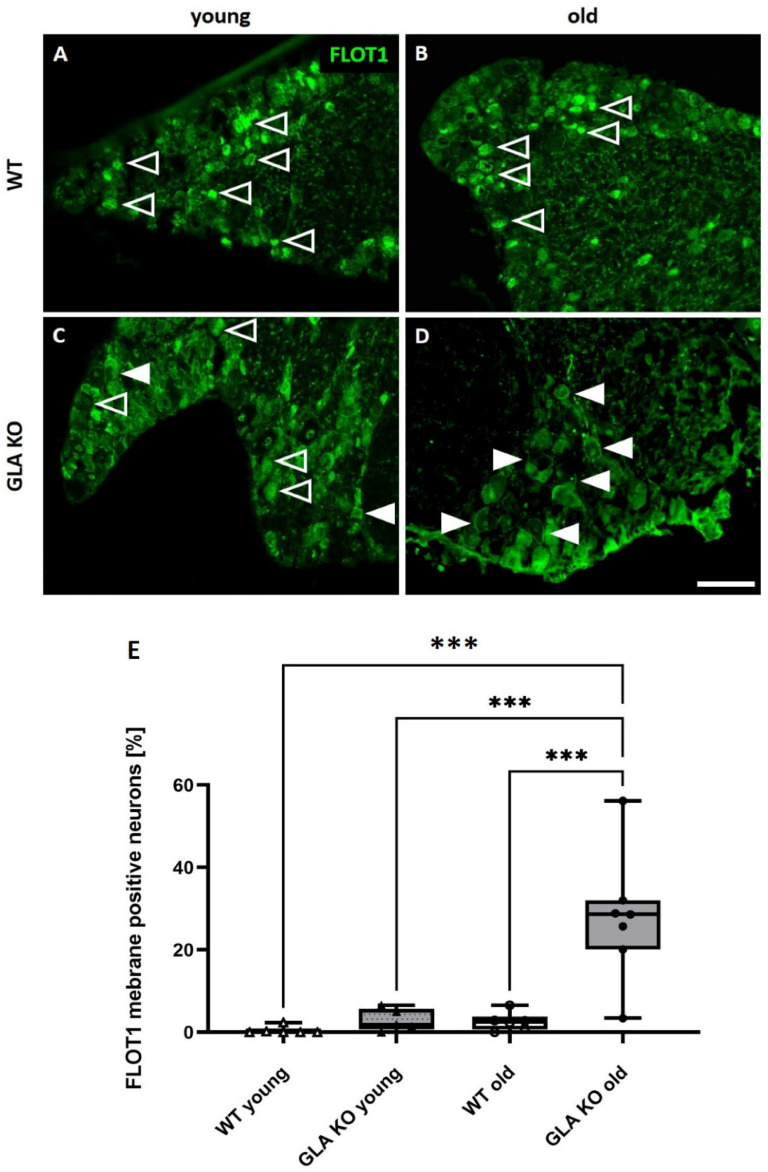
**FLOT1 distribution analysis in murine DRG neurons.** (**A**–**D**) Representative photomicrographs of FLOT1 distribution in murine DRG neurons. (**A**) Granular cytosolic FLOT1 distribution in DRG neurons of young (**A**) and old (**B**) WT mice (empty arrowheads). Mixed FLOT1 distribution represented as granular cytosolic signal (empty arrowheads) and membranous signal (full arrowheads) in DRG neurons of young GLA KO (**C**). Predominantly membranous FLOT1 distribution in DRG neurons of old GLA KO mice (**D**). Quantification of FLOT1-positive DRG neurons of young WT (△, *n* = 6); young GLA KO (▲, *n* = 5), old WT (◯, *n* = 6), and old GLA KO (●, *n* = 7) mice displaying a membranous distribution pattern (**E**). Abbreviations: DRG = dorsal root ganglia; FLOT1 = flotillin-1; GLA KO = alpha-galactosidase A knockout; WT = wildtype. Scale bar: 100 µm. *** *p* < 0.001.

Next, we investigated the distribution pattern of the lipid raft marker FLOT1, which might contribute to impaired ion channel expression, distribution, and function. We assessed the FLOT1 fluorescence signal distribution in young and old WT and GLA KO DRG neurons (Figure 7). We found a punctate cytosolic FLOT1 signal in young and old WT DRG neurons (Figure 7A,B). Young GLA KO DRG neurons displayed a mixed distribution pattern of FLOT1 represented by a punctate cytosolic and membranous signal (Figure 7C). Old GLA KO DRG neurons, in contrast, showed predominantly a membranous FLOT1 signal (Figure 7D). We further quantified FLOT1^+^ neurons showing a membranous distribution pattern in young and old WT and GLA KO DRG neurons (Figure 7E). We found a higher number of FLOT1^+^ neurons showing a membranous distribution pattern in old GLA KO DRG neurons compared to young WT and GLA KO, and old WT DRG neurons (Figure 7E, *p* < 0.001 each). There were no intergroup differences between young WT, young GLA KO, and old WT DRG regarding the number of FLOT1^+^ neurons with a membranous signal (Figure 7E, *p* > 0.05 each).

### 3.7. Old GLA KO Mice Show Mechanical Hypersensitivity and Cold Hyposensitivity Compared to WT Mice, and Age-Dependent Heat Hyposensitivity Compared to Young GLA KO Mice

**Figure 8:**
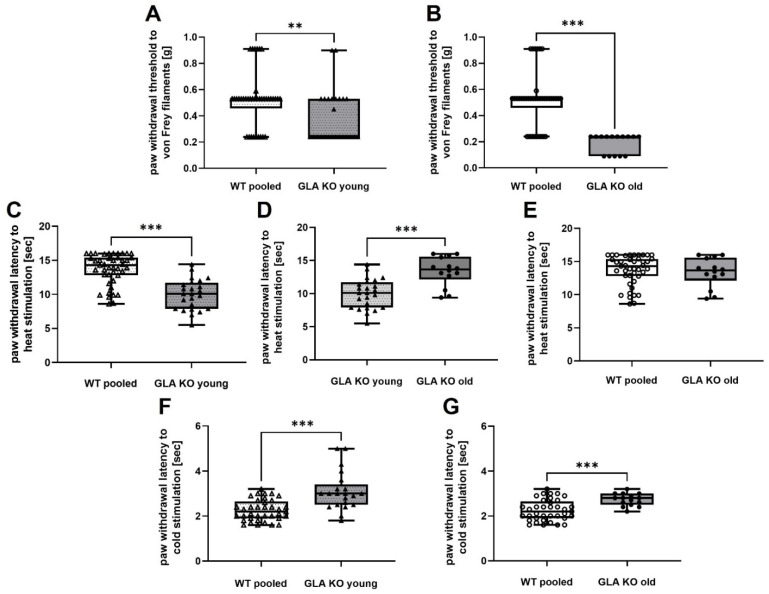
**Behavioral analysis of pooled WT mice and young and old GLA KO mice including von Frey test, Hargreaves test, and cold sensitivity test.** (**A**) Von Frey testing of pooled WT mice (△, *n* = 38) and young GLA KO mice (▲, *n* = 25). (**B**) Von Frey testing of pooled WT mice (◯, *n* = 38) and old GLA KO mice (●, *n* = 15). (**C**) Hargreaves testing of pooled WT mice (△, *n* = 43) and young GLA KO mice (▲, *n* = 24). (**D**) Hargreaves testing of young (▲, *n* = 24) and old (●, *n* = 14) GLA KO mice. (**E**) Hargreaves testing of pooled WT mice (◯, *n* = 43) and old GLA KO mice (●, *n* = 14). (**F**) Cold sensitivity testing of pooled WT mice (△, *n* = 37) and young GLA KO mice (▲, *n* = 21). (**G**) Cold sensitivity testing of pooled WT mice (◯, *n* = 37) and old GLA KO mice (●, *n* = 15). Abbreviations: g = gram; GLA KO = alpha-galactosidase A knockout; sec = seconds; WT = wildtype. ** *p* < 0.01, *** *p* < 0.001.

To match the molecular experiments with the behavioral characteristics of the GLA KO mouse model, we assessed the behavioral reactions of young and old GLA KO, and pooled WT mice after mechanical, heat, and cold stimulation (Figure 8). The von Frey test using mechanical stimulation to determine paw withdrawal thresholds at standardized von Frey filaments showed mechanical hypersensitivity in young (Figure 8A, *p* < 0.01) and old (Figure 8B, *p* < 0.001) GLA KO mice compared to pooled WT mice.

With the Hargreaves test using an IR emitter as a standardized heat source, we demonstrated that young GLA KO mice displayed heat hypersensitivity compared to pooled WT mice (Figure 8C, *p* < 0.001) and developed an age-dependent heat hyposensitivity comparing young to old GLA KO mice (Figure 8D, *p* < 0.001). Old GLA KO compared to pooled WT mice did not show differences in paw withdrawal threshold to heat stimulation (Figure 8E, *p* > 0.05).

In the behavioral test for cold sensitivity, using dry ice as a cold source, young (Figure 8F, *p* < 0.001) and old (Figure 8G, *p* < 0.001) GLA KO mice showed cold hyposensitivity compared to pooled WT mice.

## 4. Discussion

Previous studies investigated the role of Gb3, inflammation, and pain in FD patients [11,17,32] and animal models of FD [7,9,14]; however, the contribution of cellular Gb3 accumulation to immune responses and FD pain development remained elusive. Thus, we present a comprehensive study investigating a potential link between DRG tissue Gb3 accumulation and pain in the GLA KO mouse model, mimicking the molecular and behavioral hallmarks of FD [7,8,33]. We provide evidence for the dysregulation of the pro- and anti-inflammatory system and altered pain-associated ion channel expression as contributors to pain in FD. 

We investigated the Gb3 distribution in DRG neurons using STxB::555, a toxin targeting Gb3 more selectively compared to the commonly used antibody against CD77 [7,34,35]. Our results revealed an increase in Gb3 load already in the DRG of young and particularly in old GLA KO mice compared to old WT littermates (Figure 1E). As previously shown [7], the apoptosis rate of old GLA KO DRG neurons is higher compared to old WT DRG neurons. This might be caused by immune response pattern recognition receptors (PRR) against pathologic Gb3 accumulation. PRR are immune sensors expressed on several immune cells, which recognize apoptotic cells and induce phagocytosis [36].

To answer the question of whether Gb3 accumulations lead to an enhanced immune response, e.g., by increased immune cell recruitment induced via PRR activation, we quantified DRG macrophages and T-cells. In human studies, elevated numbers of macrophages were reported in the plasma of FD patients [37] compared to healthy controls. In an FD rat model, abundant macrophage and T-cell infiltration into the back skin of 90-week-old GLA KO rats was found compared to age-matched WT rats [9]. In a GLA KO mouse model crossbred with transgenic mice expressing human Gb3 synthase (GlaTg(CAG-A4GALT)), Maruyama et al. found increased numbers of F4/80^+^ macrophages in kidney tissue compared to WT mice [38,39]. We did not observe an intergroup difference when quantifying macrophages and T-cells in the DRG of old GLA KO mice compared to old WT mice (Figure 2 and Figure 3G), and counts were equally similar when focusing on the pro-inflammatory M1 macrophage subtype (Figure 3H). However, we detected lower numbers of anti-inflammatory M2 macrophages in the DRG of old GLA KO mice compared to old WT mice (Figure 3I), indicating reduced anti-inflammatory polarization of present macrophages [40]. It is known that activation of PRR leads to a suppressed immune response and thus contributes to the avoidance of an exaggerated autoimmune reaction during the phagocytic clearance of apoptotic cells [36].

Additionally, we found suppressed levels of pro- and anti-inflammatory mediators in the DRG of the GLA KO mouse model. Similar to our findings, Kummer et al. reported downregulated genes in the DRG of GLA KO mice that are involved in immune-related, autoimmune, and infection pathways [8]. Dysregulation of immune response pathways is supported by the low expression levels of the pro-inflammatory cytokine IL1b and the anti-inflammatory cytokine IL10 in the DRG of old GLA KO mice (Figure 5A,B). Interestingly, human data on pro-inflammatory cytokine gene expression levels in the peripheral blood mononuclear cells (PBMC) of male FD patients showed higher levels of tumor necrosis factor alpha (TNFa), IL1b, and toll-like receptor 4 (TLR4) as pro-inflammatory mediators and also higher gene expression levels of systemic IL4 and IL10 as anti-inflammatory mediators [11].

IL10 is further known to be upregulated via the ion channel inhibition of TRPV1 and KCa3.1 [18,19,20] and can itself downregulate NaV1.7 and NaV1.8 [24]. Further, IL10 can induce M2 polarization [41], which was reduced in our study and may be caused by downregulated IL10 gene expression levels in the DRG of old GLA KO mice.

IL1b gene expression levels are reciprocally influenced by ion channels such as TRPV1 and KCa3.1 [42,43], and by glial cell activation [44]. Thus, lower gene expression levels of the glial cell-specific marker GFAP, as reported for DRG of old GLA KO mice (Figure 5C), may further indicate an overall influence of inflammation-associated markers on ion channel gene expression, neuronal modulation, and excitability [45].

Another inflammation-associated target is LRG1, mainly contributing to inflammatory diseases such as acute appendicitis [46], inflammatory bowel disease [47], diabetic kidney disease [48], and to various cancer types [49]. LRG1 has hardly been investigated in neuropathic pain-related diseases; however, it is known to induce apoptosis via the TGF1b pathway [49]. We found reduced LRG1 gene expression in the DRG of old GLA KO mice compared to old WT littermates (Figure 5D) as a potential dysregulator of immune responses and apoptotic processes in the DRG neurons of GLA KO mice, as previously reported [7].

Next, we analyzed the gene expression levels of selected pain-associated ion channels potentially dysregulated by the imbalance of inflammation-associated targets in murine DRG and investigated further the impact on the behavioral phenotype of GLA KO mice. We found lower gene expression levels of the KCa3.1 and TRPA1 ion channels in the DRG of old GLA KO mice compared to old WT mice (Figure 6A and B, respectively), which are important contributors to neuropathic pain [50,51,52]. 

KCa3.1 strongly impacts macrophage polarization and activation [42,52,53] and can regulate IL10 levels via its inhibition [19,20,54]. While there is evidence for the bidirectional regulation of KCa3.1 and IL10, we demonstrated that KCa3.1 and IL10 gene expression levels were both reduced in the DRG of old GLA KO mice compared to old WT. Additionally, KCa3.1 blocking reduces astrocyte activation [55], supporting our results of reduced KCa3.1 and GFAP gene levels in the DRG of old GLA KO mice.

TRPA1, as a chemo-, cold-, and mechano-sensitive ion channel [22,56], is involved in the regulation of the excitatory properties of murine DRG neurons, leading to aberrant nociceptive behavior development [51]. Further, TRPA1 positively correlates with GFAP levels [21], supporting our data of reduced gene expression levels of both TRPA1 and GFAP in old GLA KO mice. Downregulation of KCa3.1 and TRPA1 gene expression can further be associated with the nociceptive behavior, as we reported for the GLA KO mouse model (Figure 8). In particular, cold hyposensitivity may be linked to reduced TRPA1 gene expression levels in old GLA KO mice [22,51,57], while the mechanical hypersensitivity reported for old GLA KO mice could be mediated via impaired TRPA1 [58,59,60], KCa3.1 [61], and NaV1.8 [62] gene expression or function. Age-dependent heat hyposensitivity in old GLA KO mice is suggested to be evoked by the loss of peripheral nerve endings, as previously reported [7]. 

While there is evidence for an interplay of several ion channels with cytokines and glial cells, such as for TRPM8 [22], CaV2.2 [23], and NaV1.8 [24,63], we did not find dysregulation of these ion channels in old GLA KO DRG tissue on a gene expression level. Dysregulated gene expression of ion channels and their functional properties may contribute to imbalanced immune responses and aberrant behavior after thermal and mechanical stimulation [7,22,23,64].

Another factor potentially contributing to ion channel impairment and thus aberrant behavior is the role of FLOT1, a lipid raft component, which is mainly involved in cellular membrane composition, trafficking, and signaling [25,26]. FLOT1 is known to influence several cellular processes, such as gene expression, receptor internalization into the cellular membrane, and T-cell activation [25,65]. Further, it was shown that FLOT1 interacts with TRPV2 channels and regulates its function in murine DRG neurons [66]. We demonstrated that old GLA KO DRG neurons show a higher number of FLOT1^+^ neurons with a membranous distribution pattern compared to young WT and GLA KO, and old WT mice (Figure 7), which might hint towards impaired internalization processes and trafficking of pain-associated ion channels such as KCa3.1 and TRPA1.

Our study has some limitations: immune response-associated genes and pain-associated ion channels were assessed mainly on a gene expression level due to limited biomaterial. Hence, we cannot draw conclusions on protein and functional properties. Nonetheless, analysis of gene expression already showed dysregulation on an immune response and ion channel expression level in the DRG of GLA KO mice. We performed our behavioral tests on native GLA KO mice without applying potential pain triggers such as physical activity since we had not found respective evidence in our previous study [31]. Regardless, we report an age-dependent pain phenotype of GLA KO mice closely mimicking the clinical FD phenotype.

**Figure 9:**
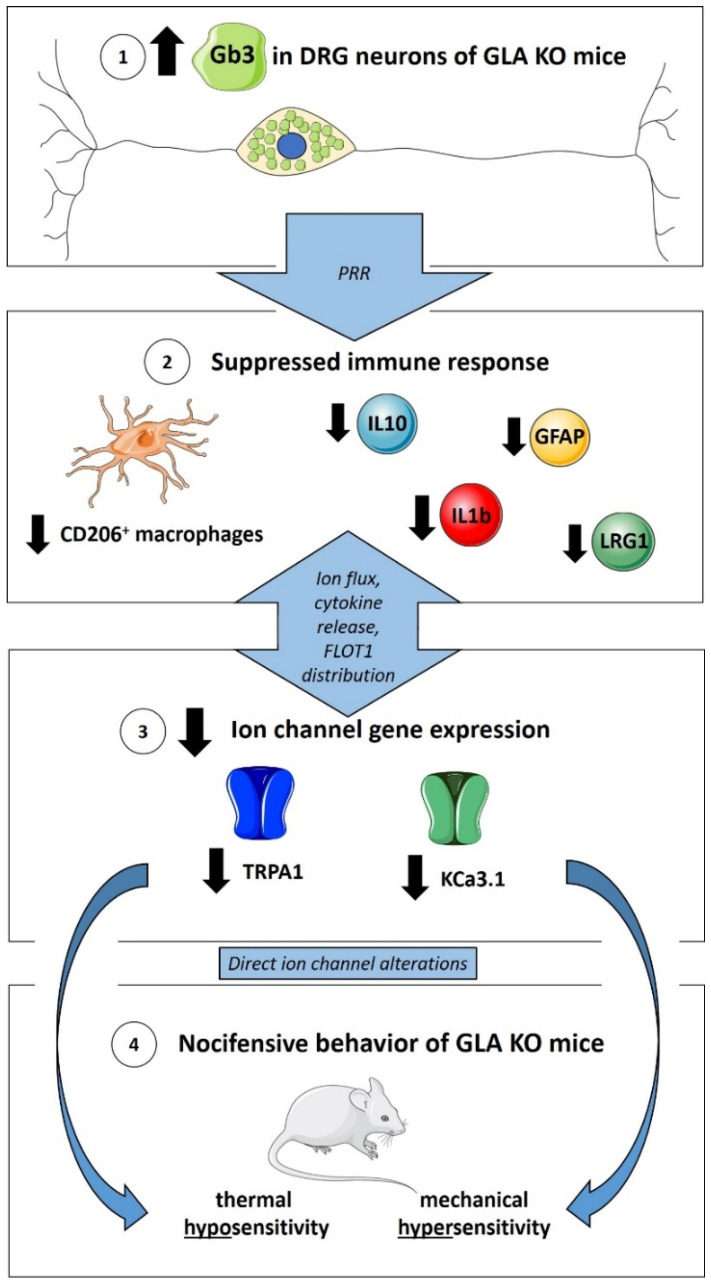
**Suppressed immune response and ion channel gene expression in murine DRG of GLA KO mice as mediators of nocifensive behavior.** ① Gb3 accumulations in DRG neurons of GLA KO mice may activate PRR on immune cells. ② Clearance of Gb3 may lead to suppressed immune response represented by reduced numbers of CD206+ macrophages and reduced gene expression of IL1b, IL10, GFAP, and LRG1, counteracting exaggerated autoimmune responses. ③ Immune response mediators might influence reciprocally pain-associated ion channel gene expression via ion flux, cytokine release, and FLOT1 distribution. ④ Reduced TRPA1 and KCa3.1 expression contributes directly to thermal hyposensitivity and mechanical hypersensitivity in GLA KO mice. Abbreviations: CD = Cluster of Differentiation; DRG = dorsal root ganglia; FLOT1 = flotillin-1; Gb3 = globotriaosylceramide; GFAP = glial fibrillary acidic protein; GLA KO = alpha-galactosidase A knockout; IL = interleukin; KCa3.1 = calcium-activated potassium channel 3.1; LRG1 = leucine-rich alpha-2-glycoprotein 1; PRR = pattern recognition receptors; TRPA1 = transient receptor potential ankyrin 1. Figure 9 contains graphics from https://smart.servier.com (last access: 24 April 2022) used under the CC BY 3.0 license.

In summary, our data support an interplay between Gb3 accumulation, imbalance in the immune response, alterations in ion channel expression, and pain-like behavior in the GLA KO mouse model (Figure 9). Our study adds to the evidence that a suppressed immune response in the GLA KO mouse model on a DRG level may play a relevant role in reduced pain-associated ion channel expression and altered nocifensive behavior. Further, we highlight potential pathomechanisms at the level of the immune response and ion channel expression, which may be potential druggable targets in novel FD pain treatment.

## Figures and Tables

**Table 1:** Used gene expression assays for single qRT PCR validation. Table includes inflammation-associated genes and ion channels.

Target Genes	Target Proteins	Assay ID
*Bcl2*	Apoptosis regulator Bcl 2	Mm00477631_m1
*C3*	Complement 3	Mm01232779_m1
*CASP3*	Caspase 3	Mm01195085_m1
*CaV2.2*	Voltage-gated calcium channel 2.2	Mm01333678_m1
*CCL2*	C-C motif chemokine 2	Mm00441242_m1
*CCL5*	C-C motif chemokine 5	Mm01302427_m1
*CD28*	Cluster of differentiation 28	Mm01253994_m1
*CD4*	Cluster of Differentiation 4	Mm00442754_m1
*CD40*	Cluster of Differentiation 40	Mm00441891_m1
*CD40lg*	Cluster of Differentiation 40 ligand	Mm00441911_m1
*CD68*	Cluster of Differentiation 68	Mm03047343_m1
*CD80*	Cluster of Differentiation 80	Mm00711660_m1
*GFAP*	Glial fibrillary acidic protein	Mm01253033_m1
*HMOX1*	Heme oxygenase 1	Mm00516005_m1
*ICAM1*	Intercellular adhesion molecule 1	Mm00516023_m1
*IKBKB*	Inhibitor of nuclear factor kappa-B kinase subunit beta	Mm01222247_m1
*IL1b*	Interleukin 1b	Mm00434228_m1
*IL4*	Interleukin 4	Mm00445259_m1
*IL6*	Interleukin 6	Mm00446190_m1
*IL10*	Interleukin 10	Mm01288386_m1
*KCa3.1*	Calcium-activated potassium channel 3.1	Mm00464686_m1
*LRG1*	Leucine-rich alpha-2-glycoprotein 1	Mm01278767_m1
*NaV1.8*	Voltage-gated sodium channel 1.8	Mm00501467_m1
*NFATC3*	Nuclear factor of activated T-cells, cytoplasmic 3	Mm01249200_m1
*NLRP3*	NACHT, LRR, and PYD domain-containing protein 3	Mm00840904_m1
*STAT3*	Signal transducer and activator of transcription 3	Mm01219775_m1
*TGF1b*	Transforming growth factor 1 beta	Mm01178820_m1
*TNFa*	Tumor necrosis factor alpha	Mm00443258_m1
*TRPA1*	Transient receptor potential ankyrin 1	Mm01227437_m1
*TRPM8*	Transient receptor potential melastatin 8	Mm01299593_m1
*VEGFa*	Vascular endothelial growth factor A	Mm00437306_m1

## Data Availability

The data presented in this study are included in the article. For further information, data are available on request from the corresponding author.

## Supplementary Materials

The following are available online at https://www.mdpi.com/article/10.3390/cells11111730/s1, Table S1: Inflammation-associated gene expression array targets. Table S2. Additional inflammation-associated gene expression assays.
