# Supplementary material for: Dysregulation of Immune Response Mediators and Pain-Related Ion Channels Is Associated with Pain-like Behavior in the GLA KO Mouse Model of Fabry Disease
Source: Cells. 2022 May 24;11(11):1730. doi: 10.3390/cells11111730 (PMC9179379; doi:10.3390/cells11111730)
Supplement: Supplementary file 1 [file cells-11-01730-s001.zip › cells-1724169-supplementary.pdf]

cells MDPI

Supplementary Materials

Supplementary Table S1. Inflammation-associated gene expression array targets. List of genes investigated with the gene expression array including targets associated with apoptosis, angiogenesis, angiotensin, endothelin, inflammation, and immune adhesion.

Target gene	Target protein	Single qRT PCR Validation (yes/no)
ACE	Angiotensin-converting enzyme	no
AGTR2	Type-2 angiotensin II receptor	no
BAX	Apoptosis regulator BAX	no
BCL2	Apoptosis regulator Bcl 2	yes
BCL2l1	Bcl-2-like protein 1	no
C3	Complement 3	yes
CCL19	C-C motif chemokine 19	no
CCL2	C-C motif chemokine 2	yes
CCL3	C-C motif chemokine 3	no
CCL5	C-C motif chemokine 5	yes
CCR2	C-C chemokine receptor type 2	no
CCR4	C-C chemokine receptor type 4	no
CCR7	C-C chemokine receptor type 7	no
CD19	B-lymphocyte antigen CD19	no
CD28	T-cell-specific surface glycoprotein CD28	yes
CD34	Hematopoietic progenitor cell antigen CD34	no
CD38	ADP-ribosyl cyclase/cyclic ADP-ribose hydrolase 1	no
CD3e	T-cell surface glycoprotein CD3 epsilon chain	no
CD4	T-cell surface glycoprotein CD4	yes
CD40	Tumor necrosis factor receptor superfamily member 5	yes
CD40lg	Cluster of Differentiation 40 ligand	yes
CD68	Macrosialin	yes
CD80	T-lymphocyte activation antigen CD80	yes
CD86	T-lymphocyte activation antigen CD86	no
CD8a	T-cell surface glycoprotein CD8 alpha chain	no
CSF1	Macrophage colony-stimulating factor 1	no
CSF2	Granulocyte-macrophage colony-stimulating factor	no
CTLA4	Cytotoxic T-lymphocyte protein 4	no
CXCL10	C-X-C motif chemokine 10	no
CXCL11	C-X-C motif chemokine 11	no
CXCR3	C-X-C chemokine receptor type 3	no
EDN1	Endothelin-1	no
FAS	Tumor necrosis factor receptor superfamily member 6	no
FASL	ADAM10-processed FasL form	no
FN1	Fibronectin	no

GZMB	Granzyme B (G, H)	no
H2-Eb1	H-2 class II histocompatibility antigen, I-A beta chain	no
Hmox1	Heme oxygenase 1	yes
ICAM1	Intercellular adhesion molecule 1	yes
ICOS	Inducible T-cell costimulator	no
IFNG	Interferon gamma	no
IKBKB	Inhibitor of nuclear factor kappa-B kinase subunit beta	yes
IL10	Interleukin 10	yes
IL12a	Interleukin 12a	no
IL12b	Interleukin 12b	no
IL13	Interleukin 13	no
IL15	Interleukin 15	no
IL18	Interleukin 18	no
IL1a	Interleukin 1a	no
IL1b	Interleukin 1b	yes
IL2ra	Interleukin 2 receptor subunit alpha	no
IL4	Interleukin 4	yes
IL5	Interleukin 5	no
IL6	Interleukin 6	yes
IL7	Interleukin 7	no
LIF	Leukemia inhibitory factor	no
LRP2	Low-density lipoprotein receptor-related protein 2	no
LY96	Lymphocyte antigen 96	no
NFATC3	Nuclear factor of activated T-cells, cytoplasmic 3	yes
NFATC4	Nuclear factor of activated T-cells, cytoplasmic 4	no
NFKB1	Nuclear factor NF-kappa-B p105 subunit	no
NFKB2	Nuclear factor NF-kappa-B p100 subunit	no
NOS2	Nitric oxide synthase 2	no
PRF1	Perforin 1	no
PTGS2	Prostaglandin G/H synthase 2	no
PTPRC	Receptor-type tyrosine-protein phosphatase C	no
SELE	E-selectin	no
SELP	Selenoprotein P	no
SKI	Ski oncogene	no
SMAD3	Mothers against decapentaplegic homolog 3	no
SMAD7	Mothers against decapentaplegic homolog 7	no
SOCS1	Suppressor of cytokine signaling 1	no
SOCS2	Suppressor of cytokine signaling 2	no
STAT1	Signal transducer and activator of transcription 1	no

STAT3	Signal transducer and activator of transcription 3	yes
STAT4	Signal transducer and activator of transcription 4	no
STAT6	Signal transducer and activator of transcription 6	no
TBX21	T-box transcription factor TBX21	no
TGF1B	Transforming growth factor 1 beta	yes
TNFa	Tumor necrosis factor alpha	yes
TNFRSF18	Tumor necrosis factor receptor superfamily member 18	no
VCAM1	Vascular cell adhesion protein 1	no
VEGFa	Vascular endothelial growth factor A	yes
18s	18s ribosomal RNA subunit	no - endogenous control
GAPDH	Glyceraldehyde-3-phosphate dehydrogenase	no - endogenous control
GUSB	Beta-glucuronidase	no - endogenous control
HPRT1	Hypoxanthine-guanine phosphoribosyltransferase	no - endogenous control

Supplementary Table S2. Additional inflammation-associated gene expression assays. Listed genes investigated with single qRT PCR analysis inluding targets associated with inflammation. Genes were not included in the gene expression array listed under Table S1.

Target gene	Target protein
CASP3	Caspase 3
GFAP	Glial fibrillary acidic protein
LRG1	Leucine-rich alpha-2-glycoprotein 1
NLRP3	NACHT, LRR and PYD domains-containing Protein 3
